# Administration of FTY720 during Tourniquet-Induced Limb Ischemia Reperfusion Injury Attenuates Systemic Inflammation

**DOI:** 10.1155/2017/4594035

**Published:** 2017-12-19

**Authors:** Anthony D. Foster, Diego Vicente, Jonathan J. Sexton, Luke Johnston, Nick Clark, Crystal Leonhardt, Eric A. Elster, Thomas A. Davis, Matthew J. Bradley

**Affiliations:** ^1^Department of Regenerative Medicine, Naval Medical Research Center, Silver Spring, MD, USA; ^2^Department of Surgery, Uniformed Services University and the Walter Reed National Military Medical Center, Bethesda, MD, USA

## Abstract

Acute ischemia-reperfusion injury (IRI) of the extremities leads to local and systemic inflammatory changes which can hinder limb function and can be life threatening. This study examined whether the administration of the T-cell sequestration agent, FTY720, following hind limb tourniquet-induced skeletal muscle IRI in a rat model would attenuate systemic inflammation and multiple end organ injury. Sprague-Dawley rats were subjected to 1 hr of ischemia via application of a rubber band tourniquet. Animals were randomized to receive an intravenous bolus of either vehicle control or FTY720 15 min after band placement. Rats (*n* = 10/time point) were euthanized at 6, 24, and 72 hr post-IRI. Peripheral blood as well as lung, liver, kidney, and ischemic muscle tissue was analyzed and compared between groups. FTY720 treatment markedly decreased the number of peripheral blood T cells (*p* < 0.05) resulting in a decreased systemic inflammatory response and lower serum creatinine levels and had a modest but significant effect in decreasing the transcription of injury-associated target genes in multiple end organs. These findings suggest that early intervention with FTY720 may benefit the treatment of IRI of the limb. Further preclinical studies are necessary to characterize the short-term and long-term beneficial effects of FTY720 following tourniquet-induced IRI.

## 1. Introduction

Acute ischemia-reperfusion injury (IRI), skeletal muscle injury, and associated secondary end organ damage can occur following revascularization of a limb following prolonged tourniquet-induced ischemia. Moreover, prolonged limb tourniquet application and the subsequent restoration of blood flow may result in inflammation-induced injury marked by the recruitment of innate immune cells, in particular, neutrophils and macrophages, and the secretion of proinflammatory cytokines and chemokines in the absence of microorganism (“sterile inflammation”) in ischemic and injured tissues [1, 2]. Spillage and amplification of these inflammatory responses systemically often lead to a complex cytokine cascade or storm that serves to perpetuate inflammatory reactions in remote organs, which can clinically manifest as multiple organ dysfunction (e.g., acute kidney injury and acute lung injury) and possibly death [3–5].

The mechanism and course of injury underlying IRI are complex, involving multiple factors and many cell types, and can vary depending on the tissues and organs affected, as well as the duration of tissue ischemia. As a rule, reperfusion of acutely ischemic tissues triggers a potent release of oxygen free radicals and cytokines which stimulate an innate immune response with subsequent leukocyte recruitment, endothelial dysfunction, and tissue damage [3]. A defining feature of IRI is the recruitment, trafficking, and accumulation of cells of the innate immune system (neutrophils and monocytes) which play an important early role in mediating tissue injury and cell destruction responses during tissue reperfusion [6]. Emerging evidence indicates that release of damage-associated molecular pattern (DAMP) molecules or alarmins at these sites prompts rapid trafficking and recruitment of T cells which serve to coordinate the local inflammatory response, supporting a pathogenic role for TCR independent activation of T cells in IRI [2, 7–10]. Therefore, therapies aimed at attenuating IRI-induced T-cell recruitment and T-cell mediated inflammatory responses may reduce the risk of multiple pathological outcomes following limb IRI.

Tissue-infiltrating lymphocytes, including T cells, were previously thought to be unimportant in the pathogenesis of IRI. However, multiple models of IRI using T cell-deficient animals have consistently demonstrated that the absence of T cells reduces the severity of IRI, while the transfer of wild-type T cells into T cell-deficient animals increases the severity of IRI (reviewed in Linfert et al.) [11]. Using a mouse model of renal IRI, CD4/CD8^−/−^ mice had reduced kidney injury and improved renal function [10]. Interestingly, CD4^−/−^ mice were similarly protected, but CD8^−/−^ mice were not [12]. Additionally, the transfer of CD4^+^ T cells from IFN-*γ*-deficient mice did not restore the normal (more severe) injury pattern nor did CD4^+^ T cells from CD28-deficient mice. Interestingly, a model of intestinal IRI supported a role for T cells in recruiting neutrophils to the site of injury as MPO activity was significantly reduced in severe combined immune-deficient mice [13]. Collectively, these findings support a critical role for CD4^+^ T cells, as well as IFN-*γ* in the pathogenesis of IRI.

FTY720 is a synthetic analog of sphingosine-1-phosphate (S1P) and functions as a superagonist at the S1P receptor, promoting internalization of the receptor which interrupts normal lymphocyte trafficking between blood and lymphoid tissues, resulting in a transient accumulation/sequestration of lymphocytes within secondary lymphoid tissues. Treatment with FTY720 promotes retention of T cells in peripheral lymph nodes by increasing the expression of integrins in high endothelial vessels, thus preventing them from trafficking to local sites of inflammation [14]. FTY720 has a demonstrated anti-inflammatory effect in models of infection [15], sepsis [16], and lung IRI [17]. Reductions in serum cytokine production were also associated with reduced vascular permeability following FTY720 treatment [16, 17]. Despite multiple animal studies demonstrating the ability of FTY720 to improve survival and/or reduce the sequelae of IRI, the effects of FTY720 on systemic inflammation and whether it ameliorates/mitigates tissue damage following limb IRI remain unclear [18–20]. Given the prominent role of inflammation in mediating the pathogenesis of IRI, our hypothesis was that FTY720 would mitigate the severity of the inflammatory response and end organ injury. As such, we sought to investigate whether the administration of FTY720 following tourniquet-induced limb skeletal muscle IRI in a rat model would dampen systemic inflammation and/or attenuate end organ injury.

## 2. Methods

### 2.1. Animals

Young adult pathogen-free male Sprague-Dawley rats (*Rattus norvegicus*; 300–350 g) were purchased from Taconic Farms (Germantown, NY). All animals were housed individually in plastic cages and kept on a 12 hr light/dark cycle with unlimited access to food (standard rodent chow) and fresh water ad libitum. They were acclimated for at least one week before experimentation. The study protocol (13-OUMD-01S) was reviewed and approved by the Walter Reed Army Institute of Research/Naval Medical Research Center Institutional Animal Care and Use Committee in compliance with all applicable Federal regulations governing the protection of animals in research.

### 2.2. Hind Limb Ischemia-Reperfusion Injury Model

Prior to experimentation, 10 animals were assigned to serve as naïve controls. The remainder of the animals were randomized into either a vehicle control group or a FTY720-treatment group and assigned for euthanasia at 6, 24, or 72 hr post-IRI (*n* = 10 for each survival time point, totaling 60 rats). All control and FTY720-treated animals received an initial intraperitoneal (i.p.) injection of tiletamine/zolazepam (Telazol) (40 mg/kg) with subsequent weight-based i.p. injections of tiletamine/zolazepam (10–40 mg/kg) as needed to achieve and maintain adequate anesthesia. The rats then underwent temporary placement of a 3M® Bummer orthodontic elastic band (4.6 mm, heavy force; 3M Unitek, Monrovia, CA) around the left hind limb proximal to the greater trochanter for 1 hr. At 15 minutes following band placement, rats received a single intravenous bolus (0.75 mL) of either FTY720 (0.3 mg/kg; Novartis Pharmaceuticals Corp, NJ) or vehicle control (50% ethanol in D-PBS).

### 2.3. Postsurgical Care

Following band removal, animals were allowed to recover and were monitored until fully awake, ambulatory, and stable. Rats were treated with buprenorphine-sustained release (1.2 mg/kg, subcutaneous, Zoopharm, Laramie, WY) postrecovery for pain relief before being returned to their home cage with full access to food and water. Rats in the 24 and 72 hr arms were monitored postrecovery at least twice a day for affected limb edema or distress by trained study team personnel.

### 2.4. Euthanasia, Blood Collection, and Tissue Harvest

Animals underwent euthanasia with pentobarbital 0.5 mL i.p., followed by exsanguination via cardiac stick. Blood was collected in EDTA tubes for clinical laboratory testing [CBC with differential, CHEM-7, alanine aminotransferase, aspartate aminotransferase (AST), alkaline phosphate (ALT), creatinine, and albumin] and polychromatic flow cytometric analysis of T cell subsets. The liver, spleen, lungs, kidneys, and ischemic limb muscle were collected for further analysis.

### 2.5. Flow Cytometry

Flow cytometry analysis was used to quantify T cells in circulation and in the spleen by CD45, *αβ*-TCR, CD4, and CD8 surface marker expression and FoxP3 intracellular expression. Approximately one-third of each spleen was gently crushed using a sterile syringe head in staining buffer (PBS+ 1% FCS+ 0.01% NaN_3_) and filtered through a 70 *μ*M nylon cell strainer (Falcon, Tewksbury, MA). Isolated splenocytes were washed and resuspended in staining buffer. Cell staining was performed using standard methods. Briefly, 10^6^ splenocytes or 100 *μ*L of whole blood was pretreated for 10 min with FC-Block [100 *μ*L in staining buffer containing 0.5 *μ*g of anti-rat CD32 (BD Biosciences, San Jose, CA)] to prevent nonspecific Fc receptor-mediated antibody binding. Cells were first stained with saturating concentrations of anti-CD45-APC-Cy7 (BioLegend, San Diego, CA), anti-CD4-V450 (BD Biosciences), anti-*αβ*-PerCP (BD Biosciences, San Jose, CA), and anti-CD8-PE-Cy7 (eBioscience, San Diego, CA) for 30 min at 4°C in the dark. Next, whole blood samples were lysed for 3 min at room temperature using ammonium chloride lysis buffer (ACK; Lonza, Walkerville, MD). All samples were washed twice and resuspended in staining buffer. Subsequently, all samples were fixed, permeabilized, and stained intracellularly with anti-FoxP3-APC (eBioscience) then washed and fixed in 1% paraformaldehyde following the intracellular staining kit provided by the manufacturer (eBioscience). Sample acquisition and multicolor data analysis were performed using a BD FACSAria™ II cell sorter and BD FACSDiva™ software (Becton, Dickinson and Company, San Jose, CA). Targeted cell populations were identified using forward versus side scattered light detection gating which is reflective of the size and granularity of cells.

### 2.6. Multianalyte Testing for Serum Inflammatory Mediators

Serum inflammatory mediators (IL-1*β*, IL-6, IL-10, IL-12p70, IFN-*γ*, IL-17A, IL-18, G-CSF, GM-CSF, MIP-1*α*, MCP-1, IP-10, MIP-2, TNF*α*, and RANTES) were measured using Luminex multianalyte profiling (Rat MILLIPLEX® MAP kit; EMD Millipore Corp, Billerica) according to the kit instructions provided by the manufacturer.

### 2.7. Tissue Histopathologic Analysis and Assessment of Pulmonary Edema

**Figure 1:**
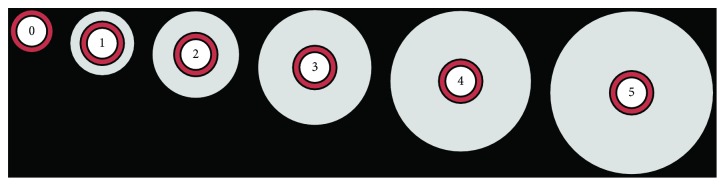
Graphic representation of perivascular edema scores 0 through 5. The white inner circle represents the vascular lumen, the red ring represents the arterial wall, and the gray outer circle represents the extent of perivascular edema surrounding the vessel.

Tissue samples were fixed in 10% buffered formalin, trimmed/processed, paraffin embedded, serially sectioned (5 *μ*m), mounted on glass slides, and then stained with hematoxylin and eosin. Stained sections were evaluated and graded by a veterinary pathologist using the grading criteria described in Tables 1–3 and Figure 1. Pulmonary edema was evaluated using wet-to-dry ratios where one unfixed lung fragment from each animal was weighed prior to and following dehydration.

### 2.8. Immunohistochemistry (IHC) of Lung MPO^+^ Leukocytes

Harvested lungs were inflated and perfused with 10% neutral-buffered formalin. Lung tissue for histopathological analysis was processed and sectioned as described above. Sections were de-paraffinized, fixed, blocked, and immuno-stained with an optimal concentration of a polyclonal rabbit anti-rat myeloperoxidase (MPO) antibody (Abcam, Cambridge, MA) followed by an optimal concentration of biotinylated secondary antibody and then a HRP-streptavidin conjugate reagent. Stain was visualized using 3,3-diaminobenzidine hydrochloride (DAB) as a chromogen. Sections were counterstained with Mayer's hematoxylin solution. Digital brightfield microscope images of immunostained slides were captured using a slide scanner and imaging system software for analysis (Image-Pro Plus versus 7.0, Media Cybernetics, Rockville, MD). Data was expressed as the mean number of MPO^+^ leukocytes from 10 random fields (200x total magnification).

### 2.9. RNA Isolation and Gene Expression

At indicated time points, small samples of the lung, liver, kidney, and ischemic muscle tissue were harvested and stored in RNALater (Ambion Inc., Austin, TX, USA) at 4°C. Tissue samples obtained from age-matched naïve uninjured rats (*n* = 5) served as control tissue. Total RNA was isolated and purified using RNeasy columns and DNase-I kits (Qiagen, Valencia, CA, USA) according to the manufacturer's protocols. Total RNA was quantified spectroscopically by using a NanoDrop 1000 (ThermoFisher Scientific, Waltham, MA), and RNA integrity/quality was assessed by microcapillary electrophoresis using an Agilent 2100 Bioanalyzer (Agilent Technologies, Santa Clara, CA). Reverse transcriptase polymerase chain reaction (RT-PCR) was performed using 1 *μ*g of RNA to synthesize cDNA. mRNA transcripts for 86 key ischemia-reperfusion-related target genes, based on an in-depth literature review, were examined by real-time PCR (QuantStudio 7 Flex Real-Time PCR System; Applied Biosystems) using a custom low-density microarray (RT^2^ Profiler PCR array, Qiagen). Gene expression was normalized to a reference gene [glyceraldehyde 3-phosphate dehydrogenase (GAPDH) or actin beta (ACTB)] and calculated relative to that of tissues collected from noninjured rats using the 2^−ΔΔCt^ method. To prepare the heat maps found in Supplementary Figure 1, the resulting gene expression data was first converted to a Log_2_ base format (to better display the range of data). Graphs were prepared using the *Matlab R2016a* (Version 9.0) program (MathWorks, Natick, MA).

### 2.10. Statistical Analysis

Continuous outcomes were compared between control and treatment groups at each time point using the Student's two-tailed *t*-test, categorical outcomes were compared using Chi-squared tests, and pathology scores were compared using the Mann–Whitney *U* test. Statistical outliers were detected using Grubb's test and removed as necessary. *P* values of ≤0.05 (two-sided) were considered statistically significant.

## 3. Results

### 3.1. FTY720 Treatment Effects on Circulating CD4^+^ and CD8^+^ T Cells following Hind Limb Ischemia-Reperfusion Injury

**Figure 2:**
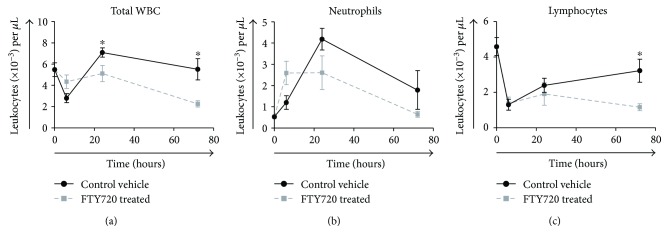
Mean peripheral blood WBC, neutrophil, and lymphocyte counts following tourniquet-induced hind limb IRI. Rats underwent hind limb ischemia by placement of a tourniquet band for 4 hr followed by reperfusion of affected limb. Rats received a single intravenous bolus of either vehicle control (•) or 0.3 mg/kg FTY720 (■) 15 min after placement of the tourniquet. Animals were euthanized at 6, 24, or 72 hr postinjury as indicated. Blood was collected by cardiac puncture for leukocyte differential counts. Data are shown as the average number of cells for each treatment group ± SEM (*n* = 7–10 rats per time point/group). ^∗^*P* < 0.05 compared with vehicle control at the same time point postinjury.

Circulating white blood cells (WBC) (1.2-fold versus time 0) and the absolute number of neutrophils (5.3-fold versus time 0) peaked at 24 hr postinjury and returned to baseline levels by 72 hr, while levels in the FTY720 treatment group were markedly lower as seen in Figure 2. The absolute number of lymphocytes decreased in both treatment groups with nadirs at 6 hr and 72 hr in the vehicle control and FTY720 treatment group, respectively.

**Figure 3:**
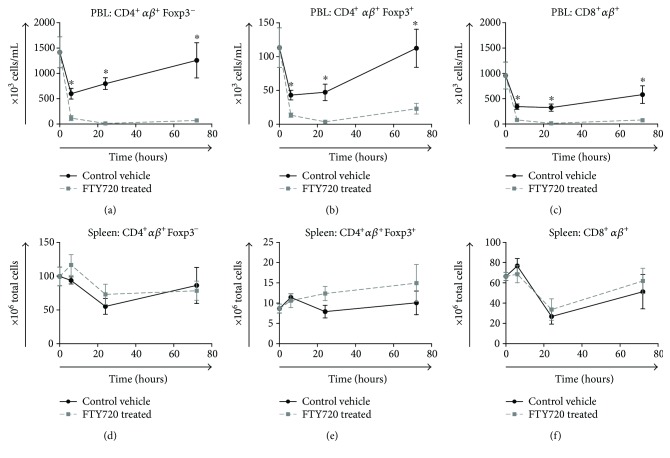
Change in the distribution of T cell subsets in the peripheral blood and spleen following tourniquet-induced hind limb IRI. Rats underwent hind limb ischemia by placement of a tourniquet band for 4 hr followed by reperfusion of affected limb. Rats received a single intravenous bolus of either vehicle control (•) or 0.3 mg/kg FTY720 (■) 15 min after placement of the tourniquet. Animals were euthanized at 6, 24, or 72 hr postinjury as indicated. Whole blood and isolated splenocytes were stained for expression of T cell markers CD4, CD8, *αβ*, and Foxp3. Using 3-color flow cytometry, gated CD4^+^*αβ*^+^Foxp3^−^, CD4^+^*αβ*^+^Foxp3^+^, and CD8^+^*αβ*^+^ lymphocytes were enumerated. Data are presented as the calculated mean ± SEM number of circulating cells per mL of blood and per spleen (*n* = 4–10 rats per time point/group). ^∗^*P* < 0.05 compared with vehicle control at the same time point postinjury.

Flow cytometric analysis of T lymphocyte subpopulations demonstrated an initial decrease in the number of peripheral blood CD4^+^*αβ*^+^ and CD8^+^*αβ*^+^ T cells in both vehicle control and FTY720 treatment groups at 6 h post band placement. However, FTY720 rats had a more profound decrease in total *αβ*^+^ T cells which remained lower than baseline values at 72 h as shown in Figure 3. Specifically, FTY720-treated rats compared to vehicle control rats had significantly fewer CD4^+^*αβ*^+^Foxp3^−^ (6 h: 117.9 × 10^3^ cells/mL versus 598.4 × 10^3^ cells/mL, *p* < 0.001; 24 h: 14.6 × 10^3^ cells/mL versus 797.9 × 10^3^ cells/mL, *p* < 0.001, 72 h: 72.3 × 10^3^ cells/mL versus 1256.7 × 10^3^ cells/mL, *p* = 0.015), CD4^+^*αβ*^+^Foxp3^+^ (6 h: 13.4 × 10^3^ cells/mL versus 43.2 × 10^3^ cells/mL, *p* < 0.001; 24 h: 3.7 × 10^3^ cells/mL versus 47.2 × 10^3^ cells/mL, *p* = 0.021, 72 h: 23.0 × 10^3^ cells/mL versus 112.5 × 10^3^ cells/mL, *p* = 0.022), and CD8^+^*αβ*^+^ (6 h: 83.2 × 10^3^ cells/mL versus 344.2 × 10^3^ cells/mL, *p* < 0.001; 24 h: 15.7 × 10^3^ cells/mL versus 325.2 × 10^3^ cells/mL, *p* = 0.007, 72 h: 77.6 × 10^3^ cells/mL versus 581.6 × 10^3^ cells/mL, *p* = 0.049) lymphocytes at all time points. In contrast, no significant differences existed between splenic CD4^+^*αβ*^+^Foxp3^−^ and CD8^+^*αβ*^+^ T cell subsets in control versus FTY720-treated groups (Figures 3(d) and 3(f)).

### 3.2. FTY720 Treatment Effects on Systemic Inflammation and End Organ Damage

**Figure 4:**
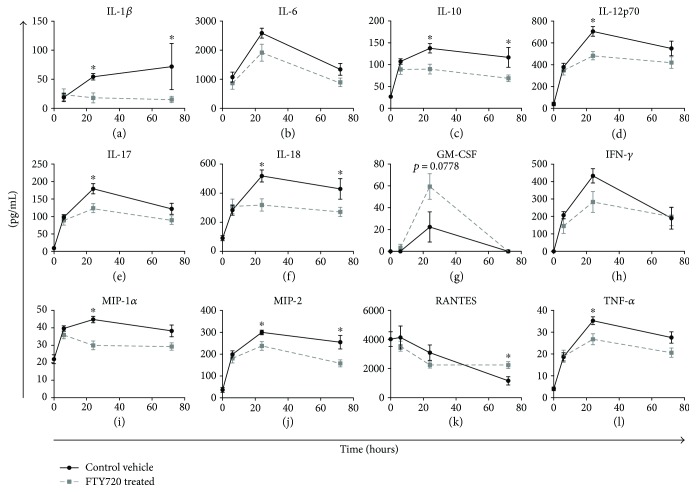
Proinflammatory cytokine and chemokine in the serum following tourniquet-induced hind limb IRI. Rats underwent hind limb ischemia by placement of a tourniquet band for 4 hr followed by reperfusion of affected limb. Rats received a single intravenous bolus of either vehicle control (•) or 0.3 mg/kg FTY720 (■) 15 min after placement of the tourniquet. Animals were euthanized at 6, 24, or 72 hr postinjury as indicated. Collected serum was assayed in duplicate for levels of circulating G-CSF, GM-CSF, MIP-1*α*, IL-1*β*, IL-6, IL-10, IL-12p70, IFN-*γ*, IL-17*α*, IL-18, MCP-1, IP-10, MIP-2, TNF*α*, and RANTES using a multiplex bead assay. Serum from naïve control animals was used for baseline measurements (time 0). Data are shown as the mean serum concentration (pg/mL) ± SEM (*n* = 7–10 rats per time point/group). ^∗^*P* < 0.05 compared with vehicle control at the same time point postinjury.

Differences in serum inflammatory mediators for HLI-injured rats treated with vehicle control or FTY720 are shown in Figure 4. The concentrations of IL-6, IL-10, IL-12p70, IL-17, IL-18, IFN-*γ*, MIP-1*α*, MIP-2, and TNF-*α* in the control group were highest at 24 hr, whereas IL-1*β* peaked at 72 hr. The concentrations of these inflammatory mediators were all markedly lower, albeit higher than baseline values, in the FTY720 treatment group 24–72 hr after IRI injury.

**Figure 5:**
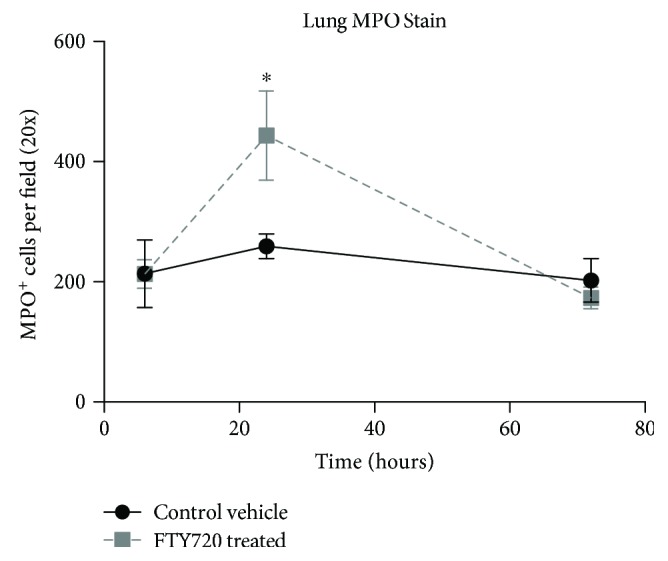
The number of lung infiltrating MPO^+^ leukocytes was significantly increased in FTY720-treated rats following tourniquet-induced hind limb IRI. Formalin fixed-paraffin embedded lung tissue was stained at 6, 24, and 72 hr for polymorphonuclear neutrophils infiltrating the lung as determined by neutrophil-specific MPO IHC staining. Digital brightfield images were taken at 200x total magnification and analyzed using Image-Pro Plus software (v7.0, Media Cybernetics). A total of 10 fields per slide/animal were analyzed. Data presented as the mean ± SEM number of MPO^+^ leukocytes per field. Vehicle control (•, *n* = 5 rats) and FTY720-treated (■, *n* = 5 rats) at the indicated time points postinjury. ^∗^*P* < 0.05 compared with vehicle control at the same time point postinjury.

No difference in pulmonary edema was observed between the FTY720 group versus vehicle control animals when comparing wet-to-dry ratios (mean ratio of 4.56 versus 4.49, *p* = 0.40; data not shown). Similarly, there was no difference in histopathologic grading of pulmonary perivascular edema between groups at 6 hr (1.44 versus 1.62, *p* = 0.77), 24 hr (1.20 versus 1.70, *p* = 0.13), or 72 hr (1.69 versus 1.71, *p* = 0.91; data not shown) time periods. However, we measured a transient yet significant increase of MPO^+^ leukocytes in the lungs of FTY720-treated animals at 24 hr (Figure 5).

**Figure 6:**
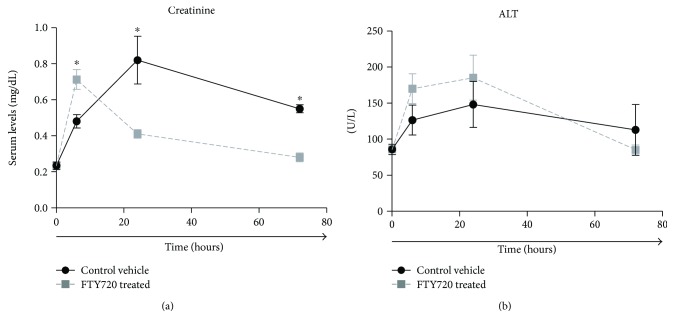
FTY720 treatment reduces kidney and liver injury after tourniquet-induced hind limb IRI in rats. Data are shown as mean ± SEM serum creatinine and alanine aminotransferase (ALT) levels for vehicle control (•, *n* = 10 rats) and FTY720-treated (■, *n* = 10 rats) rats at the indicated time points postinjury. Baseline (time 0) values represent data from serum collected from untreated naïve control animals. ^∗^*P* < 0.05 compared with vehicle control at the same time point postinjury.

Serum creatinine in the FTY720 treatment group had a higher level at 6 hr (0.71 versus 0.50 mg/dL, *p* < 0.05) but a lower level at 24 hr (0.4 versus 0.8 mg/dL, *p* < 0.05) and 72 hr (0.3 versus 0.6 mg/dL, *p* = 0.07) (Figure 6(a)). Alanine aminotransferase (ALT) levels were elevated compared to baseline in both the control and treatment groups, but there were no significant differences in this enzyme level between the two groups at any time point (Figure 6(b)).

### 3.3. FTY720 Treatment Reduces the Expression of Several Key Tissue Injury and Cell Damage-Associated mRNA Gene Transcript

**Figure 7:**
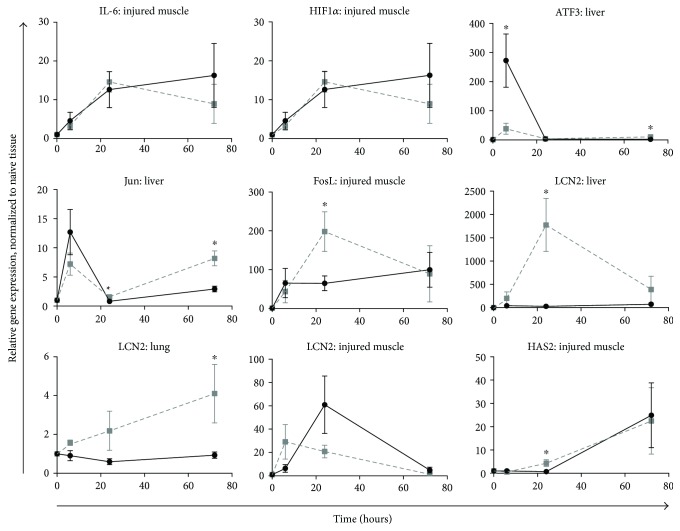
Differential expression of selected genes in ischemic muscle, liver, and lung tissue in FTY720-treated rats (■) versus vehicle control-treated rats (•) following tourniquet-induced hind limb ischemia. Tissues at the indicated time point post-IRI were stored in RNALater and subsequently processed for mRNA. cDNA conversion of 1 *μ*g of RNA was performed by RT-PCR followed by semiquantitative real-time PCR for gene expression analysis using the 2^−ΔΔCT^ method. A custom low-density array panel of 88 genes relevant to ischemia-reperfusion injury was selected for the analysis. For each tissue, gene expression was quantified relative to naïve control animals. ^∗^*P* < 0.05 compared with vehicle control at the same time point postinjury.

Semiquantitative real-time PCR was performed on mRNA isolated from ischemic muscle, liver, kidney, and lung tissues. Fold change in expression versus naïve tissue was calculated using the ΔΔCt method [21]. The resulting values were transformed to Log base 2 values (in order to better represent the range of data) and are represented in heat maps shown in Supplemental Figure 1 A–D (S1 A–D). The heat maps include data from ischemic muscle (S1A), kidney (S1B), liver (S1C), and lung (S1D). The data, organized by gene function, indicate that treatment results in several divergent patterns of expression that vary by tissue type and time point following injury. To demonstrate variance and highlight statistical significance, the differential expression of selected genes is shown in Figure 7. Compared to noninjured tissue, IL-6 gene transcripts in ischemic muscle (Figure 7(a)) were elevated in both treatment groups, with no significant differences. HIF-1*α* gene transcripts likewise increased in ischemic muscle but with no treatment effect from FTY720 compared to vehicle control (Figure 7(b)). At 6 hr, liver tissue from FTY720-treated rats displayed a 7-fold reduction in the transcript levels of the stress-induced gene ATF3 (Figure 7(c)) versus that of vehicle control-treated rats. However, this trend was reversed by 72 hr in FTY720-treated versus vehicle control-treated rats. Components of the AP-1 transcription factor were increased by treatment at different points in the study. Notably, FTY720 treatment increased the liver expression of Jun (Figure 7(d)) at 24 hr and 72 hr and also increased transcript levels of FosL1 (Figure 7(e)) in ischemic muscle at 24 h. Transcript levels of LCN2 were upregulated by FTY720 treatment in both liver (Figure 7(f)) and lung tissue (Figure 7(g)), but not in ischemic muscle (Figure 7(h)), at 24 hr. Finally, the transcript level of HAS2 (Figure 7(i)) was elevated earlier in ischemic muscle (24 hr) collected from FTY720-treated animals.

## 4. Discussion

The pathogenesis of IRI involves multiple factors that give rise to a dysregulated immune response involving both pro- and anti-inflammatory signaling pathways [11]. Reperfusion of ischemic-injured limb tissue at the time of surgical resuscitation following tourniquet removal results in the transfer of inflammatory mediators from ischemic tissues into the periphery promoting the development of a systemic inflammatory response syndrome (SIRS), extremity compartment syndrome, multiple organ dysfunction syndrome (MOD), and death [4, 22–24]. Here, we demonstrate that a single bolus of the lymphocyte sequestration drug FTY720 markedly reduces the number of circulating T cells, attenuates the production of inflammatory cytokines, and mitigates the severity of renal injury in rats subjected to tourniquet-induced limb IRI.

Lymphocyte sequestration in this model notably preceded a significant reduction in critical proinflammatory mediators in the blood including IL-1*β*, IL-10, IL-12, IL-17, IL-18, TNF-*α*, MIP-1*α*, and MIP-2 at 24–72 hrs. These findings are consistent with an active role for lymphocytes in driving systemic inflammation in this model. As expected, diverse circulating T lymphocyte cell populations, including CD4^+^*αβ*^+^Foxp3^−^, CD4^+^*αβ*^+^Foxp3^+^, and CD8^+^*αβ*^+^ subsets, were significantly reduced by FTY720 treatment. FTY720 treatment did not, however, result in appreciable alteration in the homing or egress of T cell subsets to the spleen which is consistent with other reported findings [14]. It has been similarly shown that S1P1 receptor agonists mitigate inflammatory responses in a variety of animal models [25–27]. However, it should be noted that these studies investigated inflammation resulting from antigen-specific adaptive immune responses rather than the innate (antigen nonspecific) immune response presented here. Although the mechanisms underlying the immunosuppressive effects of FTY720 in this model are not clearly defined, lymphocyte sequestration does correlate with a decrease in the production and systemic secretion of multiple inflammatory mediators. It is not apparent, from these data, if the sequestration of a specific subset of T cells or other lymphocyte population to lymphoid organs is critical to improvement in disease features.

The increased presence of neutrophils in the blood at 24 h coincided with an increased presence of MPO^+^ neutrophils in the lungs following FTY720 treatment and concomitant with T cell sequestration from the periphery. A potential mechanism of action for FTY720 in this model then is the proximal separation of nonspecifically activated lymphocytes into secondary lymphoid organs and away from neutrophils which are known to be mediators of pathogenesis in IRI. Unlike lymphocytes, FTY720 binding of neutrophil SIP receptors impairs homing to lymph nodes without impacting migration to sites of inflammation [28]. This separation of cell types implies a role for neutrophil/T cell crosstalk in IRI induction. Previous studies have similarly demonstrated through *in vitro* experiments that the presence of neutrophils cocultured with stimulated T cells increases the percentage of IL-17 and IFN-*γ*-producing T cells, while the presence of CD4^+^ T cells also activated neutrophils [28, 29]. Additionally, reduction in the expression of chemotactic proteins MIP-1*α* and MIP-2 involved in the recruitment and activation of neutrophils may infer reduced inflammatory leukocyte trafficking to sites of end organ damage downstream of reperfusion. A trauma and hemorrhagic shock study, in fact, showed pulmonary protective effects of FTY720 by limiting neutrophil priming and pulmonary microvascular dysfunction [30]. While our study did not demonstrate beneficial impact of FTY720 on pulmonary edema or lung histopathology, increased infiltration MPO^+^ leukocyte into the lungs appears to be transient, resolving by 72 hr.

Extremity IRI can result in direct renal insult through rhabdomyolysis-induced proximal tubule toxicity, tubular obstruction, and vasoconstriction as well as indirect insult through immune system activation in the kidney [19, 31–35]. While these data do not allow for speculation as to whether reduced renal injury occurs as a direct result of diminished systemic inflammation, the concurrence of this at 24 h suggests a potential link. Previously proposed explanations of FTY720-mediated renal protection also include S1P1 tubular signaling, local modulation of dendritic cell activity, increased local Treg cells, and alterations in TLR2 and TLR4 expression as demonstrated in renal ischemia-reperfusion injury studies [35, 36].

The gene transcriptional patterns from multiple tissues reveal several interesting trends in FTY720 versus vehicle control injured rats. First, expression of the gene for the proinflammatory cytokine IL-6 (Figure 7(a)) as well as the hypoxia response gene HIF-1*α* (Figure 7(b)) did not vary significantly at the site of injury. This observation is consistent with a previous study of FTY720 in a model of sepsis in which treatment reduced the serum protein expression levels of IL-6, but not the mRNA transcript levels [16]. Alternatively, it is possible that FTY720 does not mitigate the extent of the initial insult but rather attenuates the systemic response to trauma following reperfusion and is consistent with the systemic (intravenous) application of FTY720. The second notable trend was in the expression of genes that are components of, or are related to, the AP-1 transcription factor complex. This includes significant changes in the expression of Jun, ATF3, and FosL. Variable increases in the expression of gene transcripts for ATF3 and JUN were detected in the lungs and liver due to treatment. AP-1, a dimer of Jun and Fos family proteins, may provide resistance to ischemia-induced damage [37]. It is known to play an important role in the response to stress, including hypoxic stress [38]. It controls the expression of inflammatory cytokines as well as components of the apoptotic signaling pathway [39]. While the data presented here do not specify a role for AP-1-related genes following IRI and treatment with FTY720, the patterned changes in gene expression across multiple tissues do highlight a potential role that warrants further investigation. Finally, there were notable changes in lipocalin-2 (LCN2) gene expression in the muscle, liver, and lungs. LCN2 is known to have a complex role in recovery from injury and other inflammatory events [40, 41]. One study reported that LCN2 expression in macrophages had cytoprotective effects that protected against end organ damage in a model of renal IRI [40]. Conversely, other studies have shown that LCN2 promotes both neutrophil chemotaxis and adhesion, thus promoting inflammation [41]. Therefore, the source of LCN2 may be important to its function in response of ischemic injury. Collectively, the gene expression data suggest that FTY720 treatment modulates the expression of transcription factors (JUN and ATF3) and proteins that may participate in the inflammatory response (HAS2 and LCN2) at the systemic rather than local level.

The authors would like to acknowledge that this study was not designed to measure a potential mortality benefit from FTY720 infusion. In addition, clinically FTY720 is giving as a daily oral dose not as single-dose intravenous infusion. Therefore, the benefits of a prolonged anti-inflammatory effect by FTY720 may be limited in our study, although suggesting an anti-inflammatory effect of FTY720, and warrants further investigation on the short-term and prolonged effects of this treatment on both limb and multiple end organ functional recovery following tourniquet-induced IRI.

## 5. Conclusion

Our results demonstrate that treatment with FTY720 resulted in a reduction in circulating T cells and subsequent attenuation of the systemic inflammatory response. In addition, our data suggests that FTY720 may limit the extent of end organ damage following IRI through an anti-inflammatory effect. Areas of future investigation should focus on discerning the role of distinct T cell subsets and evaluation of a multifaceted approach of combining numerous agents with different mechanisms of action to attenuate inflammation, mitigate end organ damage, and ultimately improve clinical outcomes after IRI.

## Figures and Tables

**Table 1:** Grading scheme for focal and multifocal lesions.

Severity	Percent of section affected	Grade	Quantifiable finding
Normal	0%	0	No foci
Minimal	<5%	1	1-2 foci
Mild	5–25%	2	3–6 foci
Moderate	25–50%	3	7–12 foci
Marked	50–75%	4	>12 foci
Severe	75–100%	5	Diffuse

**Table 2:** Grading scheme for pulmonary perivascular edema.

Severity	Extent of edema	Grade	Quantifiable finding
Normal	None	0	None
Minimal	Very small amount	1	Up to 2 times the average width of the arterial wall
Mild	Small amount	2	3 to 5 times the average width of the arterial wall
Moderate	Moderate amount	3	6 to 7 times the average width of the arterial wall
Marked	Large amount	4	8 to 10 times the average width of the arterial wall
Severe	Very large amount	5	>10 times the average width of the arterial wall

**Table 3:** Grading scheme for focal and multifocal skeletal muscle lesions.

Severity	Percent of skeletal muscle section affected	Grade	Quantifiable finding
Normal	0	0	No foci
Minimal	<5%	1	1-2 foci
Mild	5–25%	2	3–6 foci
Moderate	25–50%	3	7–12 foci
Marked	50–75%	4	>12 foci
Severe	75–100%	5	Diffuse

Scoring methodology: 1: assess skeletal muscle at 200x magnification; 2: for every field, estimate the percent of the visual field containing necrotic myocytes (0%, 25%, 50%, 75%, or 100%); 3: average 10 fields; 4: convert the percentage to a grade based on the grading scheme.

## Supplementary Materials

Supplementary materials Supplemental Figure S1: Differential expression heat maps from tissue in FTY720-treated versus vehicle control treated rats following tourniquet-induced hind limb ischemia. Tissues at 6, 24, and 72 hr point post IRI were stored in RNALater and subsequently processed for mRNA. cDNA conversion of 1μg of RNA was performed by RT-PCR followed by semi-quantitative real-time PCR for gene expression analysis using the 2^−ΔΔCT^ method. Data was then transposed into a Log_2_ Base format to better display the range of expression observed. A custom low density array panel of genes relevant to ischemia reperfusion injury was selected for the analysis. For each tissue, gene expression was quantified relative to naïve control tissue. Supplemental Figure S1-A: Ischemic Muscle Gene Expression, Supplemental Figure S1-B: Kidney Gene Expression, Supplemental Figure S1-C: Liver Gene Expression, Supplemental Figure S1-D: Lung Gene Expression.
