# Supplementary material for: Administration of FTY720 during Tourniquet-Induced Limb Ischemia Reperfusion Injury Attenuates Systemic Inflammation
Source: Mediators Inflamm. 2017 Dec 19;2017:4594035. doi: 10.1155/2017/4594035 (PMC5749296; doi:10.1155/2017/4594035)
Supplement: Supplementary materials — Supplemental Figure S1: Differential expression heat maps from tissue in FTY720-treated versus vehicle control treated rats following tourniquet-induced hind limb ischemia. Tissues at 6, 24, and 72 hr point post IRI were stored in RNALater and subsequently processed for mRNA. cDNA conversion of 1μg of RNA was performed by RT-PCR followed by semi-quantitative real-time PCR for gene expression analysis using the 2−ΔΔCT method. Data was then transposed into a Log2 Base format to better display the range of expression observed. A custom low density array panel of genes relevant to ischemia reperfusion injury was selected for the analysis. For each tissue, gene expression was quantified relative to naïve control tissue. Supplemental Figure S1-A: Ischemic Muscle Gene Expression, Supplemental Figure S1-B: Kidney Gene Expression, Supplemental Figure S1-C: Liver Gene Expression, Supplemental Figure S1-D: Lung Gene Expression. [file 4594035.f1.pptx]

## Slide 1
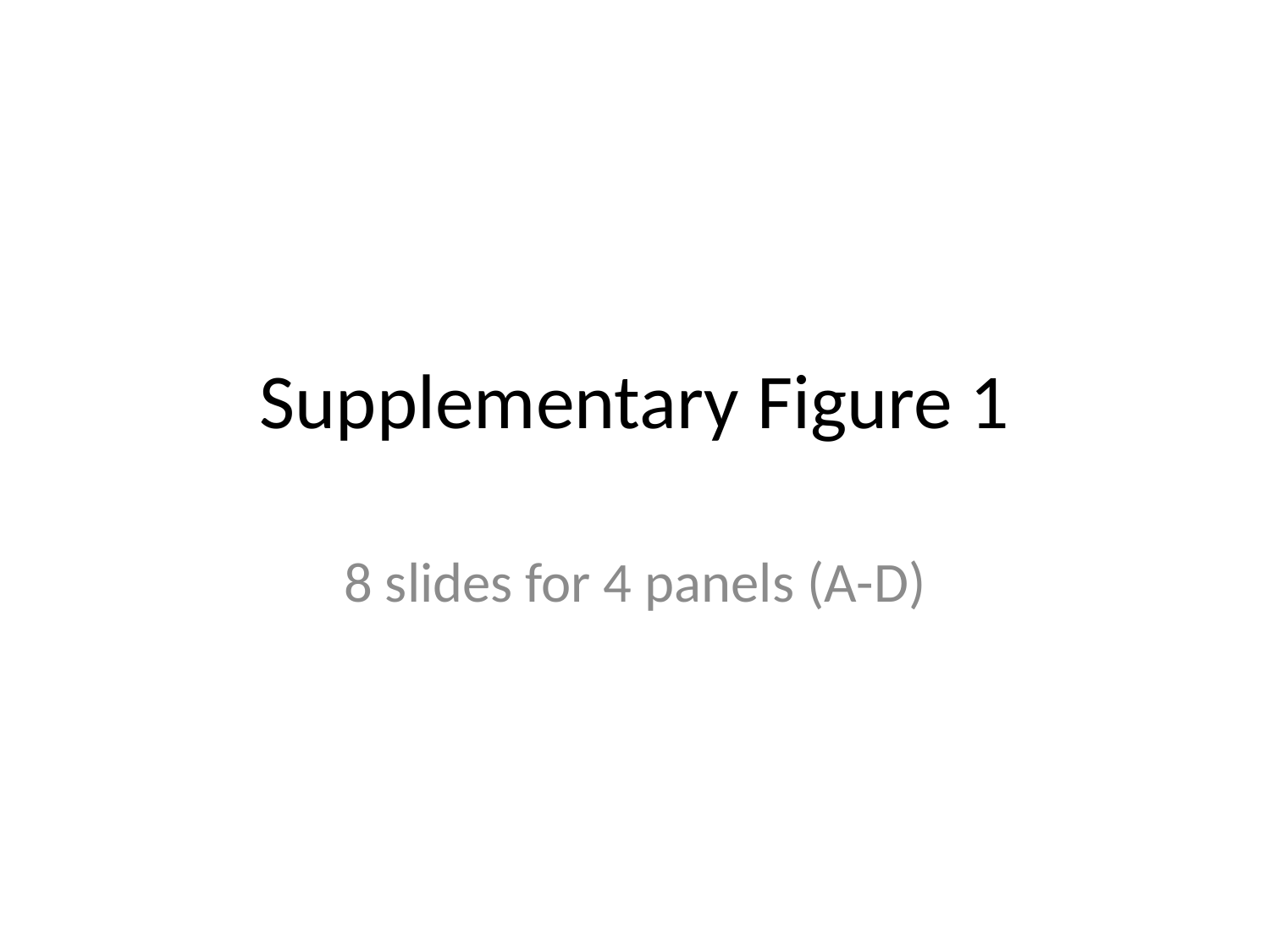

# Supplementary Figure 1
8 slides for 4 panels (A-D)

## Slide 2
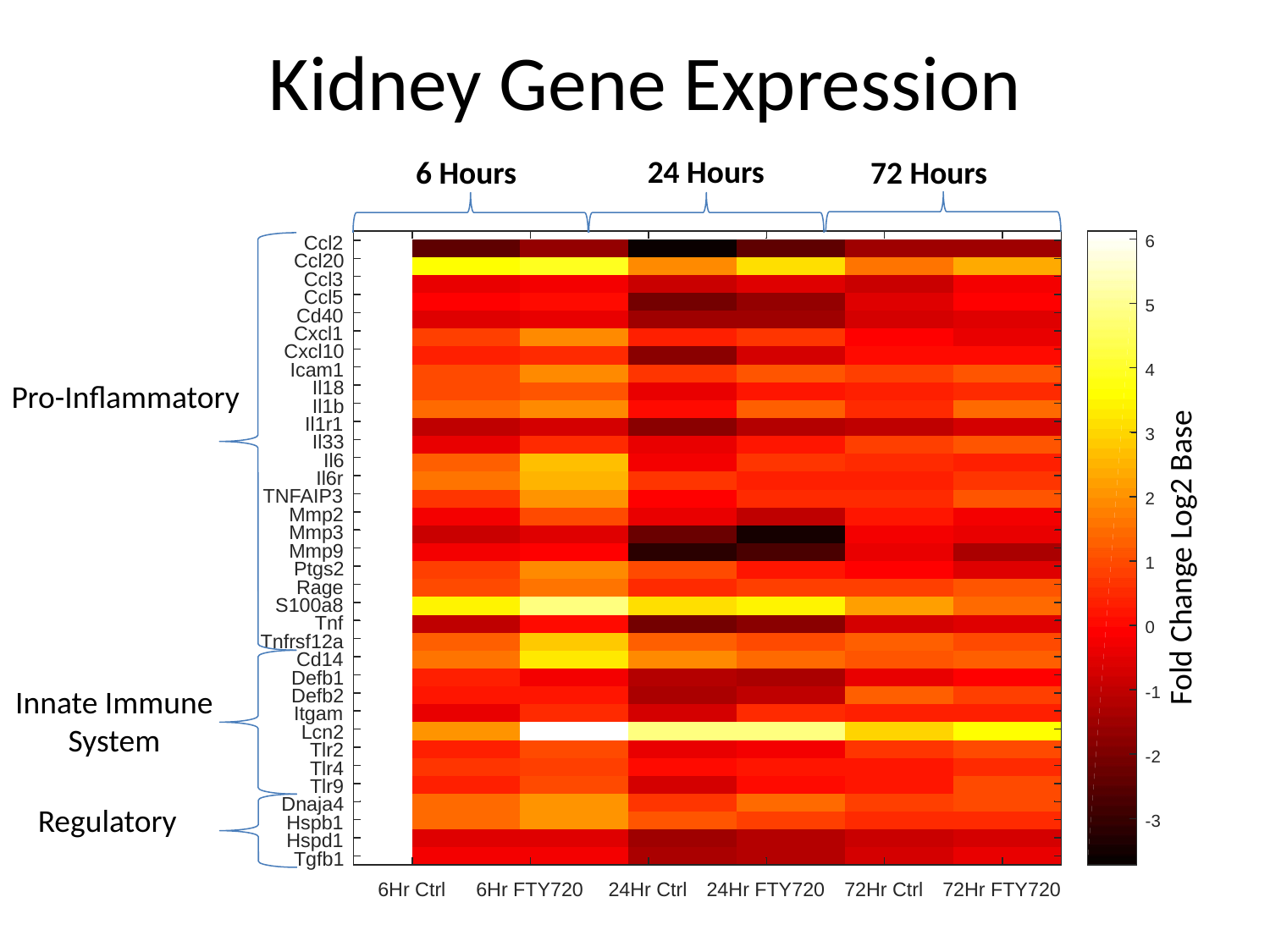

Kidney Gene Expression
24 Hours
6 Hours
72 Hours
Pro-Inflammatory
Fold Change Log2 Base
Innate Immune
System
Regulatory

## Slide 3
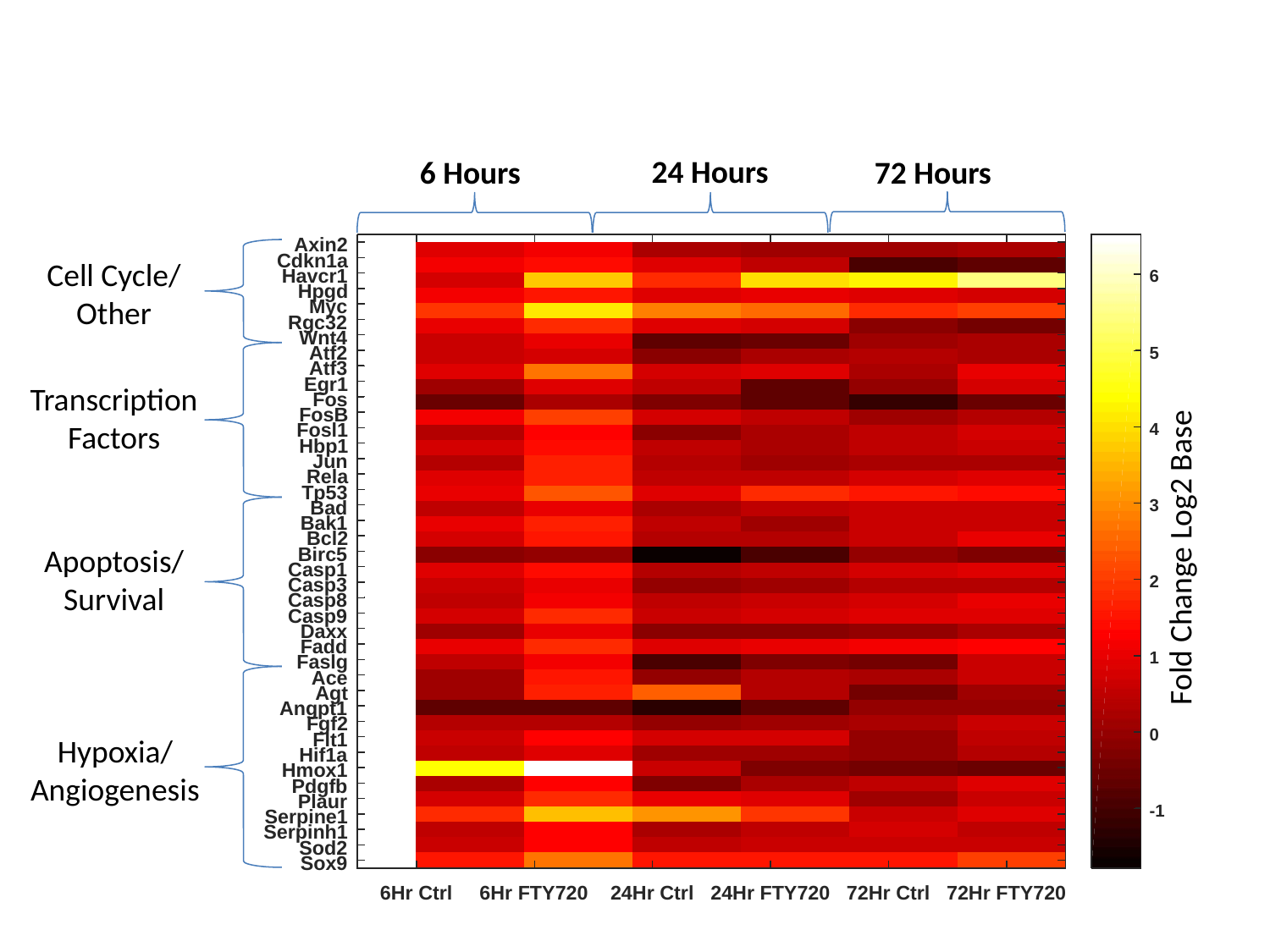

24 Hours
6 Hours
72 Hours
Cell Cycle/
Other
Transcription
Factors
Fold Change Log2 Base
Apoptosis/
Survival
Hypoxia/
Angiogenesis

## Slide 4
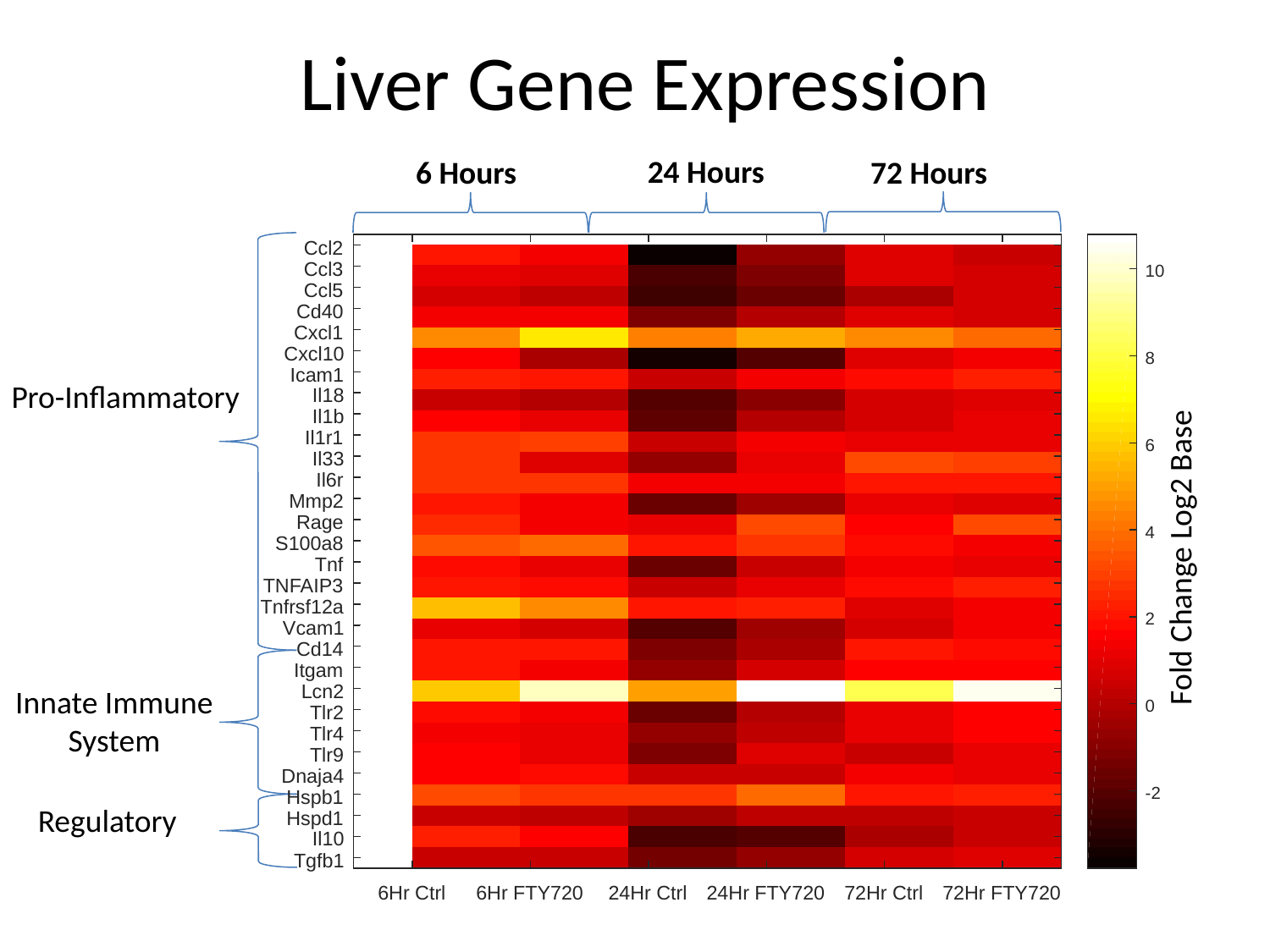

Liver Gene Expression
24 Hours
6 Hours
72 Hours
Pro-Inflammatory
Fold Change Log2 Base
Innate Immune
System
Regulatory

## Slide 5
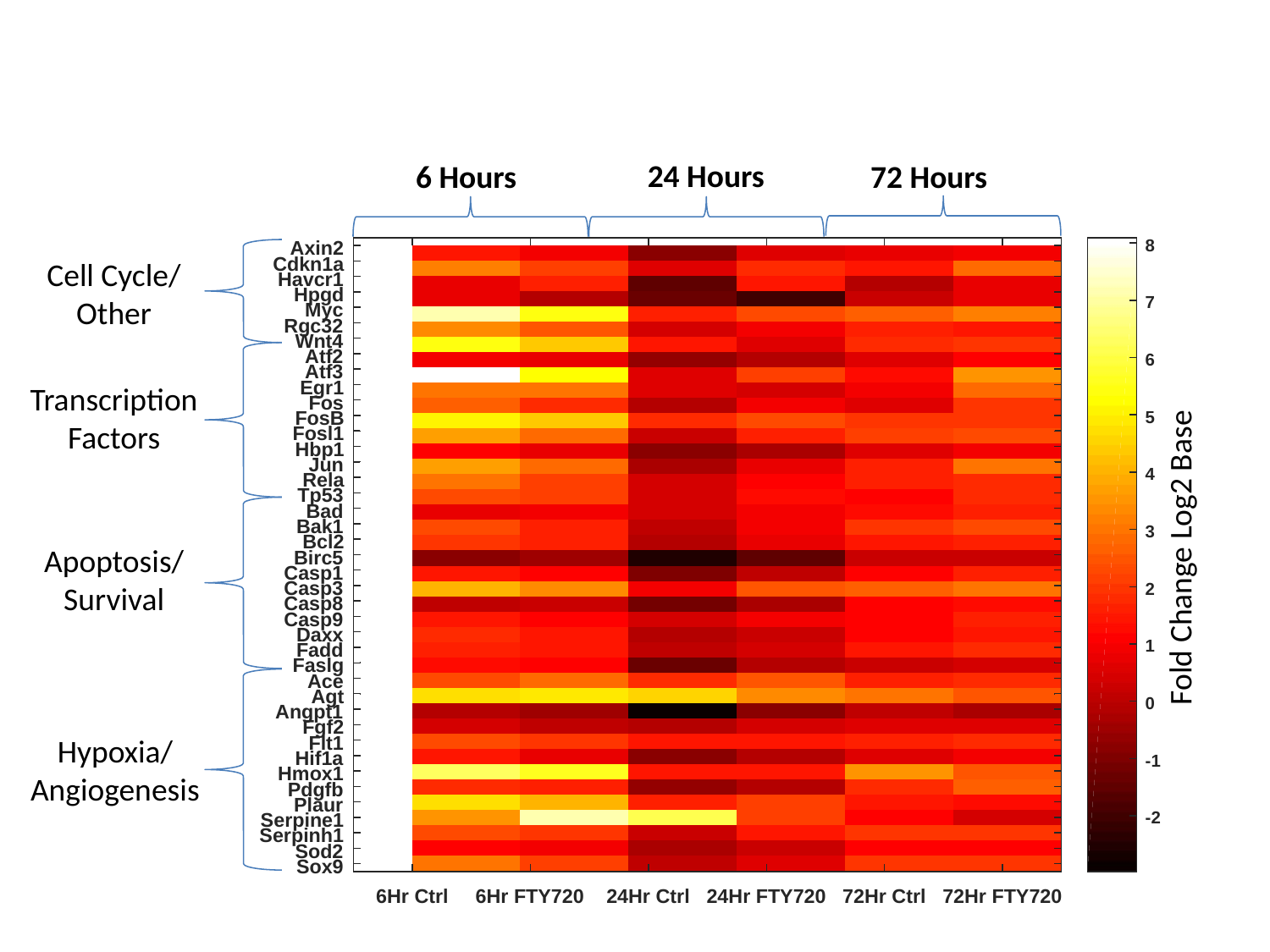

#
24 Hours
6 Hours
72 Hours
Cell Cycle/
Other
Transcription
Factors
Fold Change Log2 Base
Apoptosis/
Survival
Hypoxia/
Angiogenesis

## Slide 6
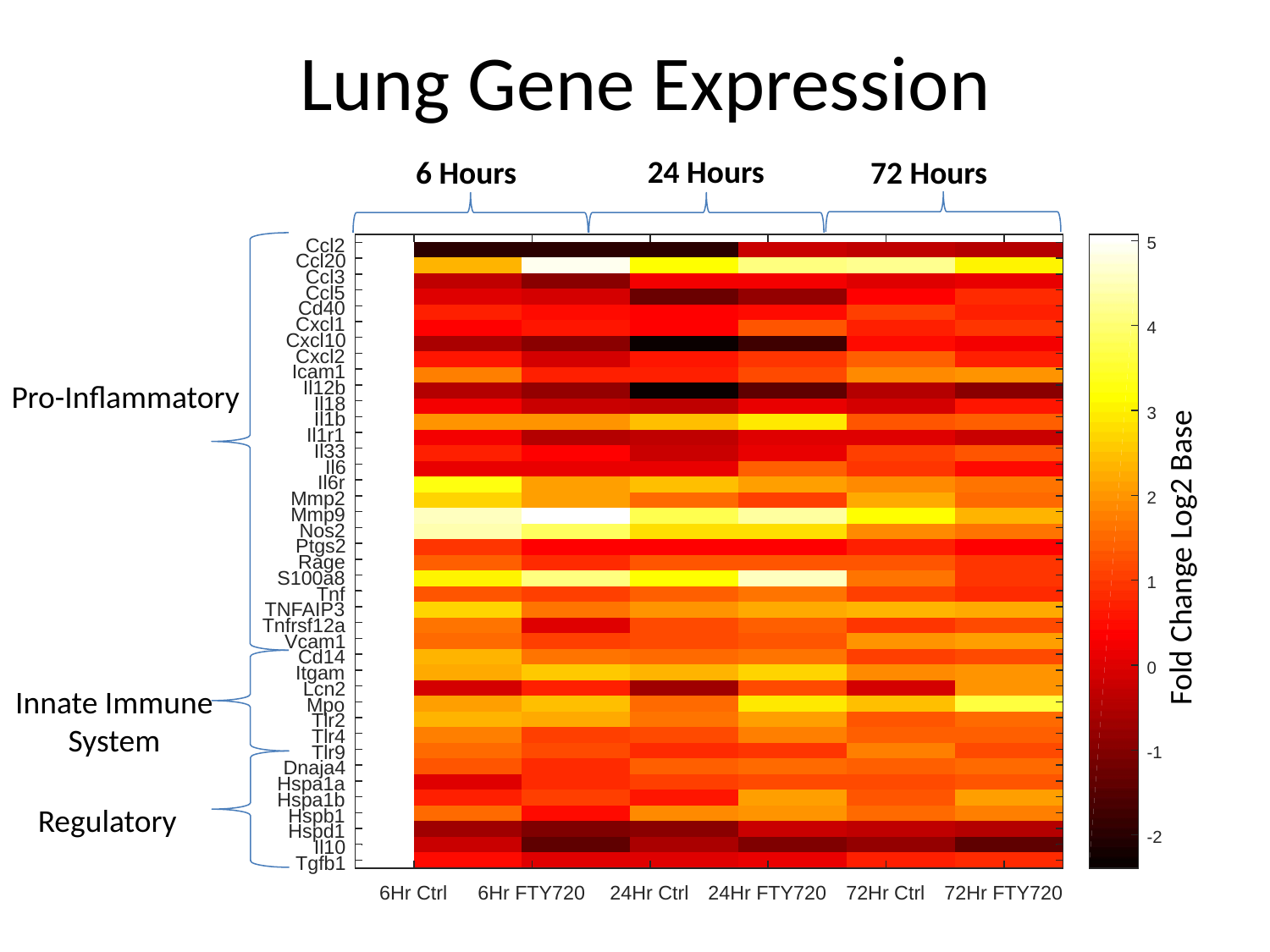

Lung Gene Expression
24 Hours
6 Hours
72 Hours
Pro-Inflammatory
Fold Change Log2 Base
Innate Immune
System
Regulatory

## Slide 7
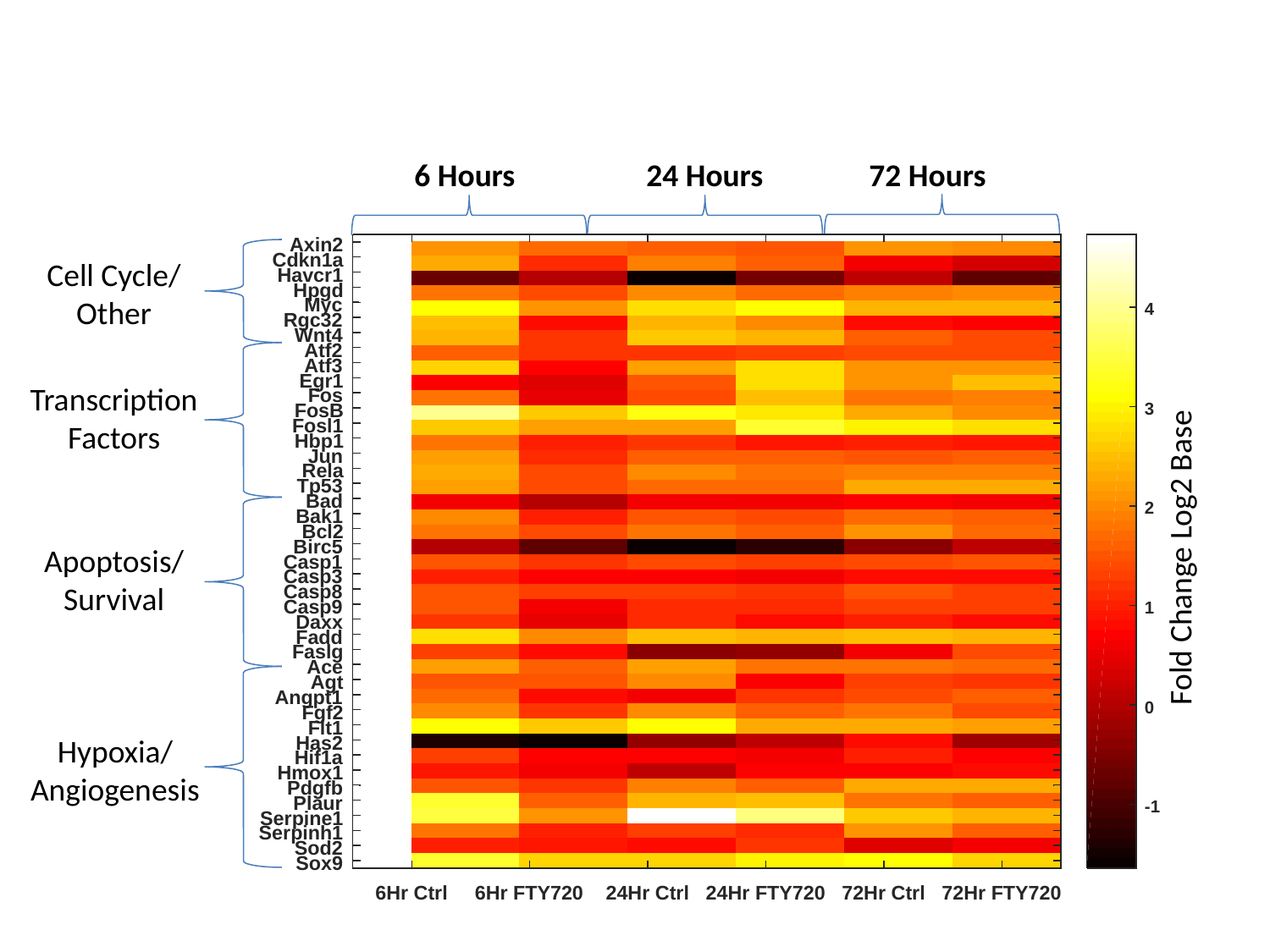

#
24 Hours
6 Hours
72 Hours
Cell Cycle/
Other
Transcription
Factors
Fold Change Log2 Base
Apoptosis/
Survival
Hypoxia/
Angiogenesis

## Slide 8
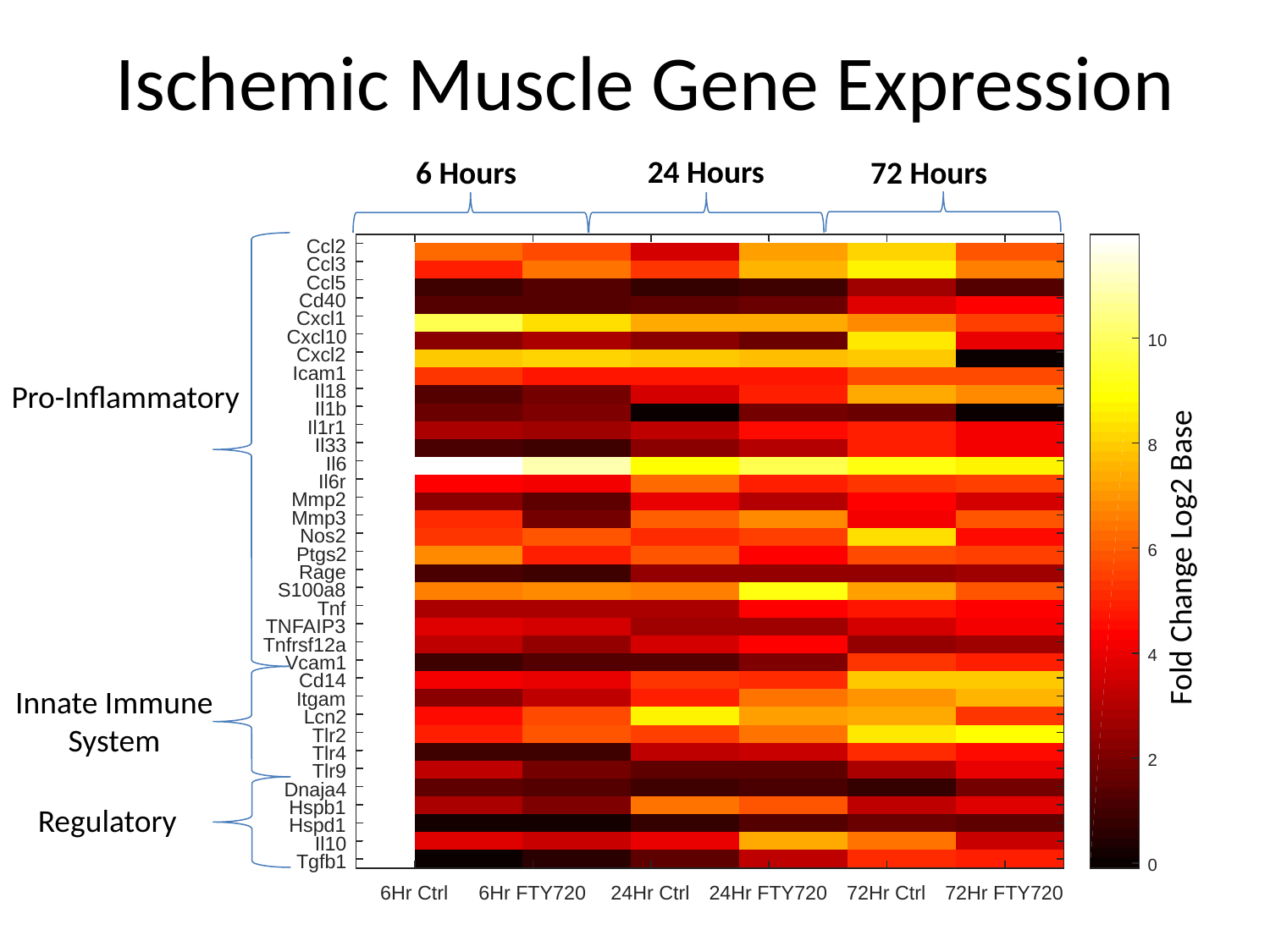

Ischemic Muscle Gene Expression
24 Hours
6 Hours
72 Hours
Pro-Inflammatory
Fold Change Log2 Base
Innate Immune
System
Regulatory

## Slide 9
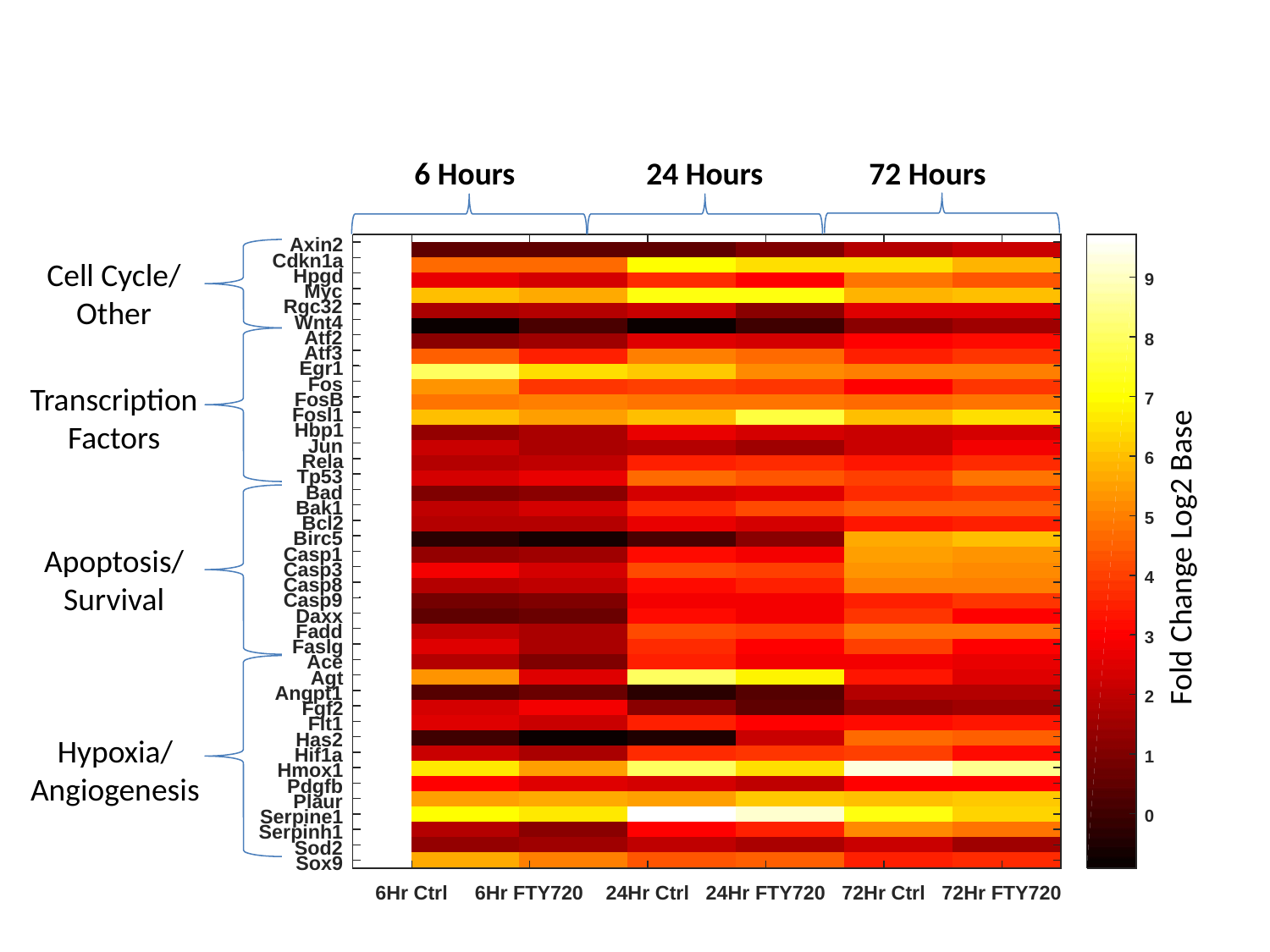

#
24 Hours
6 Hours
72 Hours
Cell Cycle/
Other
Transcription
Factors
Fold Change Log2 Base
Apoptosis/
Survival
Hypoxia/
Angiogenesis
